# Optimal cDNA microarray design using expressed sequence tags for organisms with limited genomic information

**DOI:** 10.1186/1471-2105-5-191

**Published:** 2004-12-07

**Authors:** Yian A Chen, David J Mckillen, Shuyuan Wu, Matthew J Jenny, Robert Chapman, Paul S Gross, Gregory W Warr, Jonas S Almeida

**Affiliations:** 1 Department of Biostatistics, Bioinformatics, and Epidemiology, Medical University of South Carolina, Charleston, SC, USA; 2 Department of Biochemistry and Molecular Biology, Medical University of South Carolina, Charleston, SC, USA; 3 Marine Biomedicine and Environmental Science Center, Medical University of South Carolina, Charleston, SC, USA; 4 South Carolina Department of Natural Resources, Marine Resources Research Institute, Charleston, SC, USA

## Abstract

**Background:**

Expression microarrays are increasingly used to characterize environmental responses and host-parasite interactions for many different organisms. Probe selection for cDNA microarrays using expressed sequence tags (ESTs) is challenging due to high sequence redundancy and potential cross-hybridization between paralogous genes. In organisms with limited genomic information, like marine organisms, this challenge is even greater due to annotation uncertainty. No general tool is available for cDNA microarray probe selection for these organisms. Therefore, the goal of the design procedure described here is to select a subset of ESTs that will minimize sequence redundancy and characterize potential cross-hybridization while providing functionally representative probes.

**Results:**

Sequence similarity between ESTs, quantified by the E-value of pair-wise alignment, was used as a surrogate for expected hybridization between corresponding sequences. Using this value as a measure of dissimilarity, sequence redundancy reduction was performed by hierarchical cluster analyses. The choice of how many microarray probes to retain was made based on an index developed for this research: a sequence diversity index (SDI) within a sequence diversity plot (SDP). This index tracked the decreasing within-cluster sequence diversity as the number of clusters increased. For a given stage in the agglomeration procedure, the EST having the highest similarity to all the other sequences within each cluster, the centroid EST, was selected as a microarray probe. A small dataset of ESTs from Atlantic white shrimp (*Litopenaeus setiferus*) was used to test this algorithm so that the detailed results could be examined. The functional representative level of the selected probes was quantified using Gene Ontology (GO) annotations.

**Conclusions:**

For organisms with limited genomic information, combining hierarchical clustering methods to analyze ESTs can yield an optimal cDNA microarray design. If biomarker discovery is the goal of the microarray experiments, the average linkage method is more effective, while single linkage is more suitable if identification of physiological mechanisms is more of interest. This general design procedure is not limited to designing single-species cDNA microarrays for marine organisms, and it can equally be applied to multiple-species microarrays of any organisms with limited genomic information.

## Background

Expression microarrays are powerful tools for human disease diagnosis, prognosis and treatment [1] offering unparalleled insight into the function of the entire genome and the dynamic interactions among genes. The ability of microarrays to identify gene expression signatures, specific subsets of genes that respond to particular stimuli, make them valuable tools for characterizing organisms' response to environmental conditions and host-parasite interactions. This method relies on organisms as sentinel markers of environmental changes. Since aquaculture marine species are easy to keep in a captive environment, they can be used as convenient sentinels by profiling their physiological responses. An efficient and economic method to quantify their physiological responses is to collect the expressed sequence tags (ESTs) with the purpose of constructing cDNA microarrays, which can be used to screen their transcriptomes. Therefore, several pilot studies have been initiated in economically important marine species to generate genomically enabled tools for the purpose of elucidating the role of biological and environmental factors in ultimately determining the difference between survival, morbidity and mortality [2-4]. The growing need for a marine functional genomics approach using microarrays bespeaks a general-purpose cDNA microarray probe selection tool to identify which ESTs to spot on the microarray from large collections of ESTs with unknown functions and variable redundancies.

The two most widely used expression microarray systems are oligonucleotide and cDNA microarrays. Oligonucleotide microarrays are generated by chemically synthesizing short oligo probes (20–70 bp) onto the slides [5]. In contrast, cDNA microarrays are created by spotting long strands of amplified cDNA sequences (e.g., the expressed sequence tags) [6]. In this paper, the sequences spotted on the arrays are referred to as "probes." Although many algorithms have been developed for selection of oligonucleotide [7-11] or gene-specific probes [12,13], only one application was found by the authors for cDNA microarray probe selection [14]. However, this algorithm was designed specifically for organisms with extensive genomic data, not for the organisms with limited genomic information.

In the absence of cDNA microarray probe selection algorithms, EST selection for spotting on microarrays has been approached using various informal methods. These methods included spotting ESTs without sequencing information, spotting only sequenced ESTs with annotations, or forcing the selection on gene-oriented clusters [15]. The choice of method typically reflects cost/benefit ratios and the stage of development of the EST collection. A comprehensive review of microarray probe selection can be found in Tomiuk and Hofmann [16]. Gene or transcript oriented clusters are generally formed by gene indexing projects, such as TIGR [17,18], Stack [19], or Unigene [20]. Gene indexing projects involve three general steps. First, the quality control step filters out contaminating sequences such as vector or bacterial sequences. Second, ESTs are partitioned into smaller clusters, often using the hierarchical single-linkage method with an arbitrarily chosen cut-off threshold [21,22]. Finally, although not all projects include a assemblage step, sequences are often assembled into contigs using existing software, such as CAP3 [23] or PHRAP [24].

In this study, we propose a probe selection procedure for cDNA microarray that tracks both sequence redundancies and functional representativeness of the selected probes in an integrated sequence diversity plot (SDP). SDP includes a sequence diversity index (SDI) to measure the sequence similarities within EST clusters quantitatively. The issue of how many probes are sufficiently representative for all collected ESTs is approached in a manner similar to the choice of dimensions to retain in principle component analysis (PCA). This approach reflects the fact that there is no definitive right answer to the question [25]; the number of "clusters" of ESTs may vary as the stringency of microarray hybridization condition changes. All collected ESTs are automatically annotated using Gene Ontology [26] terms, and then a unique probe GO index (UPGI), a functional index, was devised to access functionally how representative the selected probes are. This integrated and flexible method using SDP allows users to decide which clustering method and stringency to use when designing a cDNA microarray for organisms with limited genomic information based on their logistical constraint and experimental purposes. A small data set of ESTs was used to test this algorithm so that the detailed results of this algorithm could be examined.

## Results

**Figure 1:**
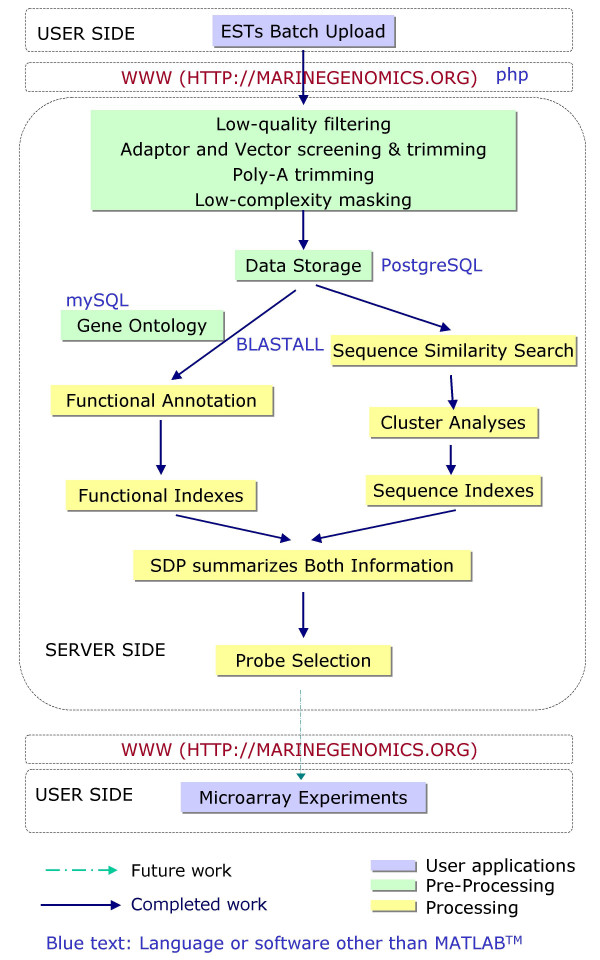
**Schematic diagram of the optimal cDNA microarray probe selection from expressed sequence tags (ESTs) for marine organisms. **The methods of this study were mainly implemented using Matlab™ and other languages or software (labelled in blue).

**Figure 2:**
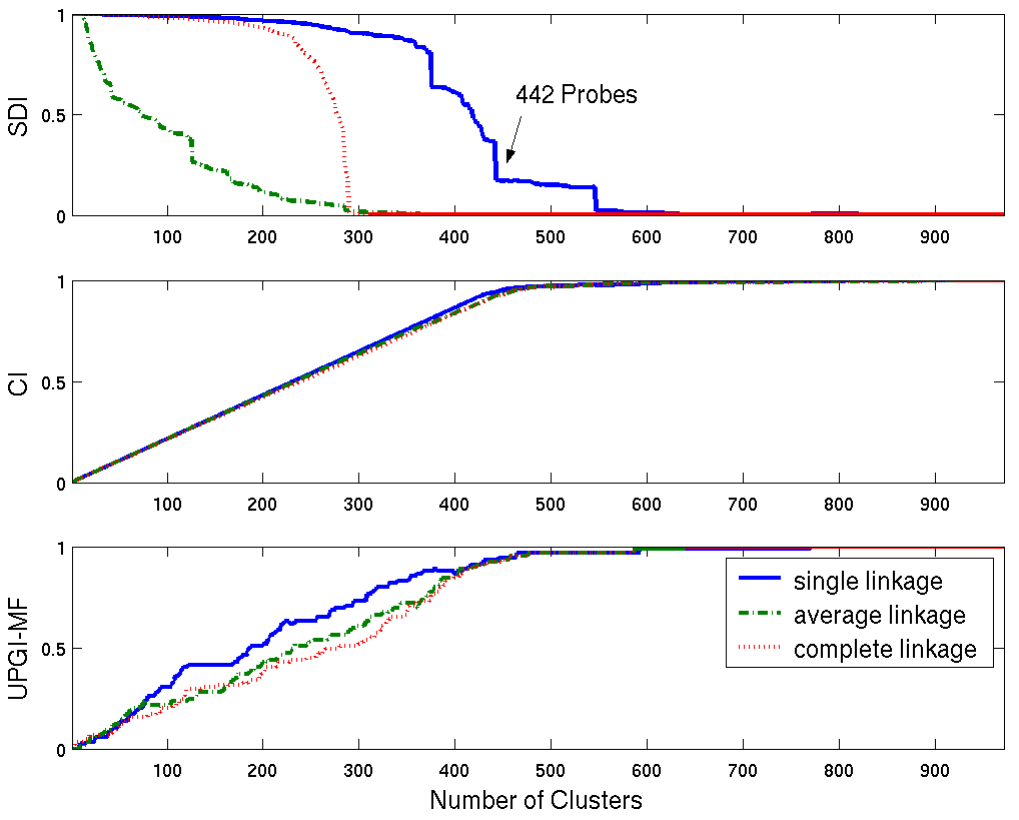
**Sequence Diversity Plot (SDP) includes both sequence diversity and probe functional representativeness **SDP summarized three indexes [the sequence dissimilarities index (SDI; Eq. (1)), the sequence contiguity index (CI; Eq. (2)), and the unique probe GO index (UPGI; Eq. (3)) in molecular function domain] among three clustering linkage methods (single, average and complete linkage methods). All indexes range between zero and one in a linear scale in this figure. Sequence diversity decreases as the number of clusters (number of selected probes) increases; functional representativeness increases as the number of probes selected increase.

A small data set of 1047 ESTs from Atlantic white shrimp (*Litopenaeus setiferus*) from the Marine Genomics website [27] was analyzed. After pre-processing, 971 sequences longer than 100 bp were further used in the analysis (details see methods; Figure 1). The ESTs were progressively grouped using different hierarchical linkage methods from 1 to n (n = 971) clusters (details see methods). The sequence diversity plot (SDP) summarizes sequence properties within clusters and the functional representativeness of the selected probes using three indexes: the sequence diversity index [SDI; Eq. (1)], the contiguity index [CI; Eq. (2)], and the unique probe GO index [UPGI; Eq. (3)] (Figure 2).

### Sequence diversity index (SDI) measures within-cluster sequence dissimilarity

This index is the ratio of within-cluster sequence dissimilarities to the total sequence dissimilarity when m clusters are formed (*m *= 1,2,...*n*):


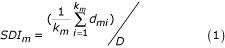


where *d*_*mi *_is the distance (dissimilarity), the E-value from blast result (details see methods), between the i^th ^pair of sequences for a total *k*_*m *_pairs of within-cluster comparisons when *m *clusters are formed. D is defined as 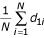, the average distance of the total N pair-wise distances among all n sequences (where 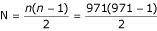 in this data set).

### Contiguity index (CI) measures the sequence contiguity within clusters

The within-cluster sequence contiguity is evaluated using CAP3 [23], commonly used sequence assembly software (see methods). The number of putative unique genes, denoted as PG_m_, is the sum of the number of assembled contigs and singlets (single sequences, which cannot be assembled with any other sequences) when m clusters (*m *= 1,2...n) are formed. The contiguity index (CI) at a given number of clusters (*m*) is defined as the inverse of the average number of putative genes per cluster, which equals the number of clusters per gene:


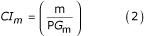


This index reflects how contiguous the sequence members are within a cluster. Maximum value of CI is 1 when all the members are contiguous (one cluster per gene).

### Unique probe Gene Ontology (GO) index (UPGI) measures functionally how representative the selected probes are

**Figure 3:**
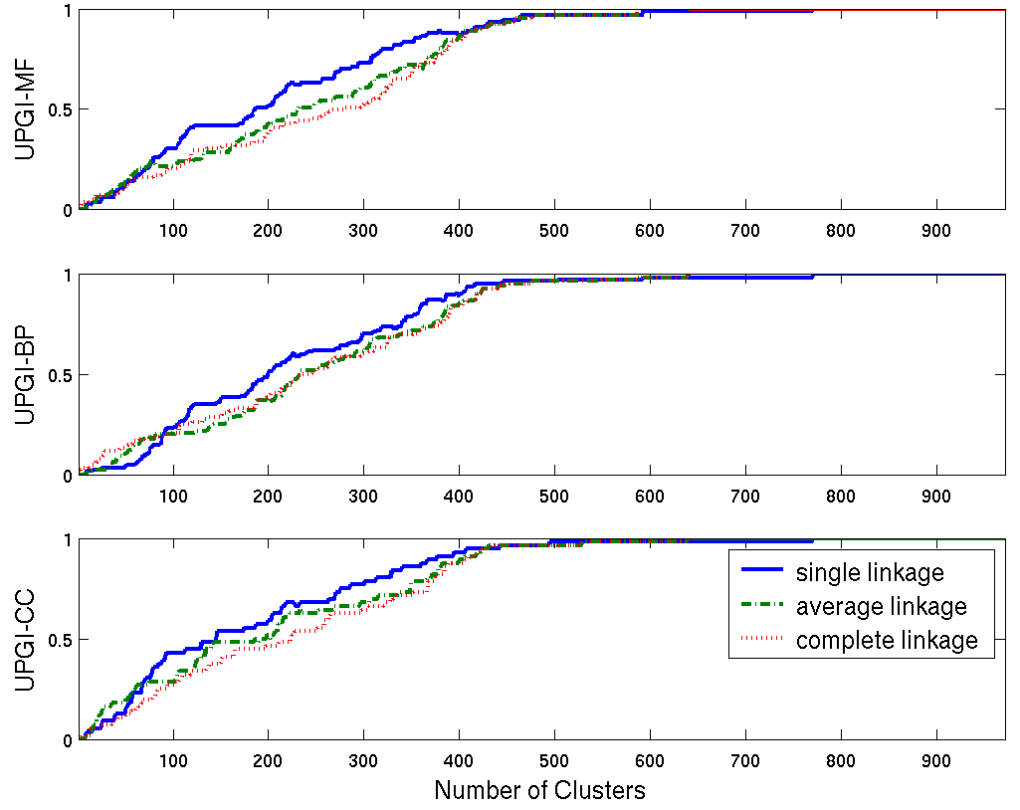
**unique probe Gene Ontology (GO) index (UPGI) in three GO domains: molecular function, biological process and cellular component. **Comparison of the unique probe GO index (UPGI) in three GO domains: molecular function, biological process, and cellular components among three linkage methods (single, average linkage, and complete linkage methods). The probes selected using single linkage were functionally more unique in all three domains than the ones selected using average or complete linkage methods when selecting middle range of number of probes (60 – 442 probes).

The unique probe GO index (UPGI) when *m *clusters of ESTs are formed is defined as the number of unique GO terms associated with all *m *probes (m = 1, 2...n) divided by the number of the GO terms associated with all *n *sequences (n = 971).


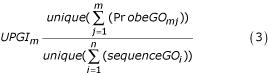


where *ProbeGO*_*mj *_is the number of unique GO terms associated with the probe representing the *j*^*th *^cluster when *m *clusters are formed and *sequenceGO*_*i *_is the number of GO terms of the *i*^*th *^sequence. This index measures functionally how representative the selected probes are among all functionally unique sequences in the entire EST collection. Three UPGIs are calculated for three GO domains, respectively: molecular function (UPGI-MF), biological process (UPGI-BP), and cellular components (UPGI-CC) (Figure 3; see more about Gene Ontology in methods).

### Sequence diversity plot (SDP) used as an aid to decide how many probes to spot on microarray

The dissimilarities among sequences within a cluster, measured by SDI, decrease as total number of the clusters increases; sequences within a cluster share higher similarity as the number of clusters formed increases (Figure 2).

From the collection of 971 *Litopenaeus setiferus *ESTs, the first break point of SDI using single linkage method was 442 clusters (Figure 2). An elbow (bend) in SDI, analogous to an elbow of scree plot of the principle component analysis (PCA), indicates that the remaining within-cluster diversity is very low after this number of clusters formed [25]. The selected probes presented 93% unique molecular functions, 94% unique biological processes, and 96% unique cellular components when 442 clusters were formed using single linkage method (Figure 3).

**Figure 4:**
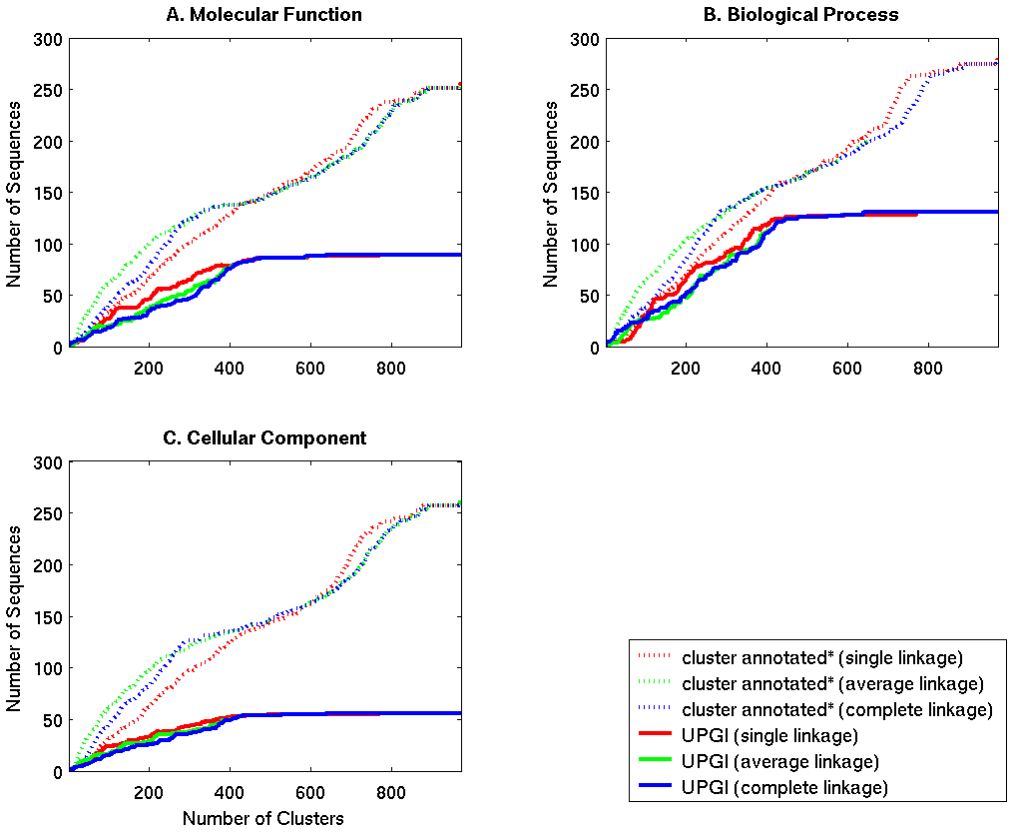
**Summary of annotated EST clusters and unique representativeness of the selected probes in three GO domains: molecular function, biological process and cellular component. **EST clusters contain at least one annotated sequences (noted as "cluster annotated*" in the legend) and unique annotations of selected microarray probes in each of the three Gene Ontology (GO) domains. (**A**) Molecular Function (MF) (**B**) Biological Process (BP) and (**C**) Cellular component (CC). Although fewer annotated EST clusters (number of clusters containing at least one annotated sequences) were formed using single linkage method compared to those selected using the other two linkage methods given a fixed number of cluster within the middle range (~60–442 clusters), more functionally unique probes were selected among the formed clusters by single linkage method

Other amalgamation algorithms produced clusterings with different properties. The average and complete linkage methods reduced the sequence dimensionality more efficiently than that by using the single linkage method (Figure 2). For the complete linkage method, the break point was observed at 289 clusters, at which, the selected probes represented only 50% of unique molecular functions while the selected probes represented 71% of unique molecular functions using single linkage, and 56% using average linkage (Figure 2). The probes selected using single linkage were functionally more unique in all three domains (molecular functions, biological processes, and cellular components) than the ones selected using average or complete linkage methods (Figure 3). Exceptions to this rule were found when very small (<60 clusters) or large (>442 clusters) numbers of probes were selected. The functional representativeness of the probes at very high or low ends (<60 or >442 clusters) was comparable using any of the three linkage methods. When 442 probes were selected, 93 – 95% unique biological process, ~92% within-cluster biological process, and ~96% unique cellular component was represented by the selected probes (Figure 3). Although fewer annotated EST clusters (number of clusters containing at least one annotated sequences) were formed using single linkage method compared to those selected using the other two linkage methods given a fixed number of cluster within the middle range (~60–442 clusters), more functionally unique probes were selected among the formed clusters by single linkage method (Figure 4).

**Figure 5:**
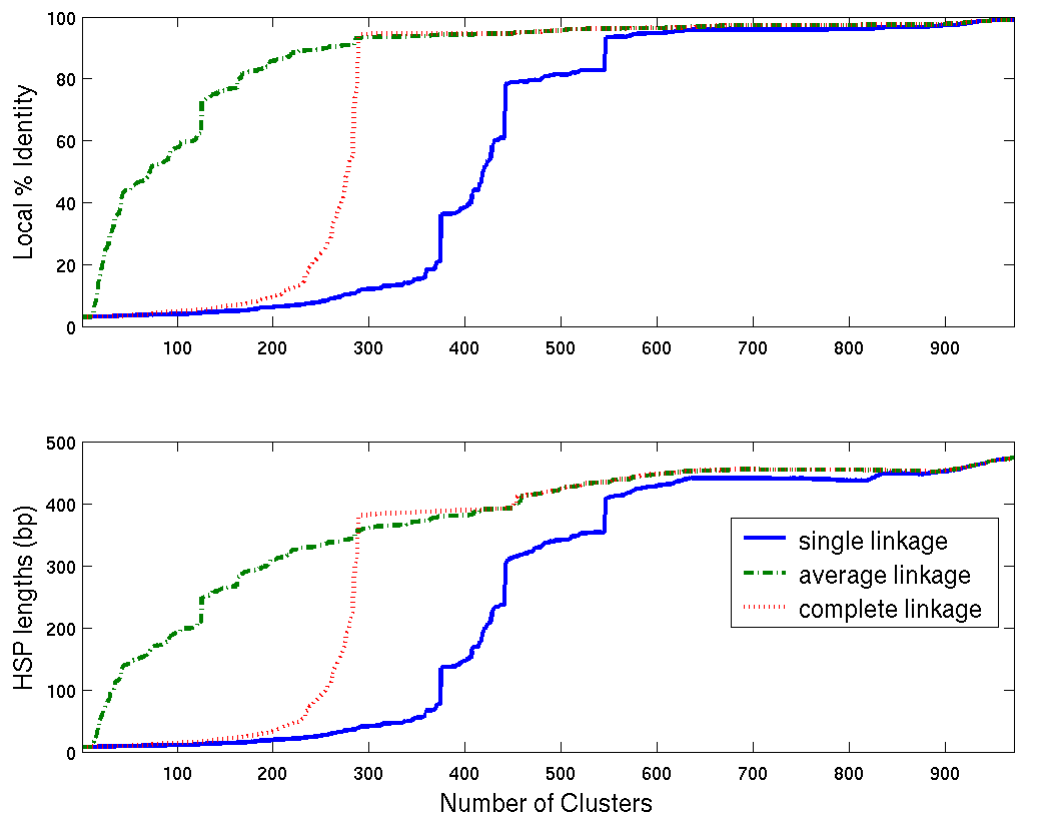
**Average within cluster percent identity and the lengths of high scoring pair (HSP) segments throughout clustering process. **The percentage identity and lengths of HSP further confirmed the observations in Figure 2 that sequences within a cluster formed using single linkage could potentially be fragments of same gene/transcript, but fragments of sequences might not overlap that average within-cluster percent identity is lower when using single linkage method. The average percent identity is the highest when using average linkage method as expected.

Contig assemblage using CAP3 yields a similar result as that of cluster analysis using the single linkage method (Figure 2). A total of 461 putative genes was generated using sequence assembly software CAP3 without partitioning the sequences into subgroups (by cluster analysis). These putative unique genes included 356 singlets (single ESTs) and 110 assembled contigs. This result followed closely the result of cluster analysis with single linkage method, which indicated 442 clusters. The EST members in each putative gene were in general agreement with the result of single-linkage cluster analysis with some exceptions. For example, sequence 59 (Penaeidin 2), sequence 10 (Penaeidin 3a), and sequences 177 (Penaeidin 3c) were not assembled into any contigs using CAP3, but they were clustered together when 422 clusters were formed using single linkage method. These sequences share high similarities and high percent identities (E-values < 10^-37^; Table 1), and they are likely to hybridize with each other. Probes selected using clustering methods reflect the hybridization potential compared to the assembly approach. Some sequences, on the other hand, were not clustered into a group although they could be assembled into one putative contig. For instance, sequences 79 and 158 were not clustered in a group because the overlapping segment is marginally short (61 bp/64 bp identical) and this segment is composed of low-complexity sequences (31 pairs of GA repeats, which were masked when using BLAST). The different characteristics of three linkage methods could be further illustrated by local sequence percent identity and the lengths of high scoring pair segments (HSP) (Figure 5). Sequences within a cluster formed using single linkage method do not always have to overlap with each other as long as the distances between some of the "linking sequences" are short (the similarities are high). That is, the fragmented ESTs could be "linked" by fragmented (or incomplete sequenced) ESTs and the average within-cluster percent identity is not necessary high when using the single linkage method (Figure 5). The sequences within same clusters using the average linkage methods, as expected, have the highest average percent identity (before all three methods converge around 545 clusters).

Sequence contiguity assessed by CAP3 (Eq. (2)) has shown similar results observed using the probe functional index, UPGI (Eq. (3); Figure 2). Clusters formed using the single linkage method contained slightly more contiguous EST members while the other two linkage methods generated fewer contiguous sequences in the mid range (Figure 2). Similarly, when the number of clusters was either very low or high, the results were comparable.

**Figure 6:**
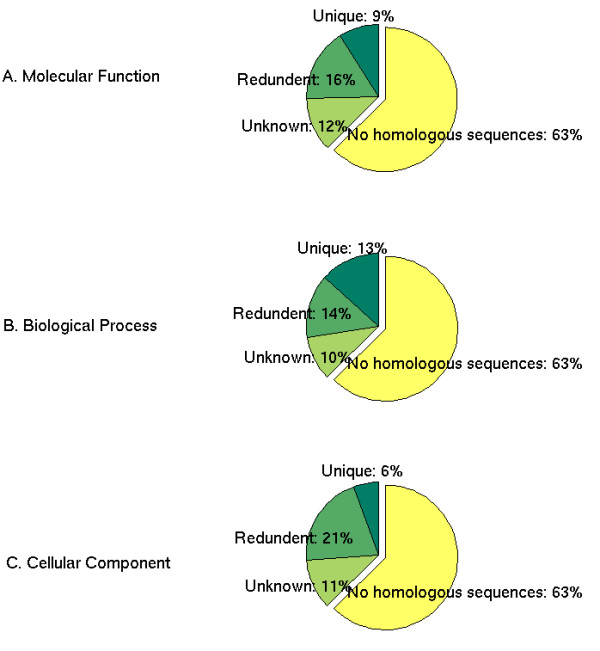
**Summary of uniquely annotated and unannotated sequences in three Gene Ontology (GO) domains. **The percentage of unique annotated sequences, "redundant" annotated sequences, and sequences have annotations in some of the three GO domains, but not in the particular domain of interest, and sequences have no similar gene products found in the Gene Ontology (GO) database (BlastX E-value was set at 10^-6^) among 971 *L. setiferus *ESTs. Three GO domains are (**A**) molecular function, (**B**) biological process and (**C**) cellular component.

ESTs were annotated based on Gene Ontology (GO) terms (details see methods). Three types of functionally unassigned sequences were generated through the GO annotation process: the first type was the sequences having no similar sequences found in the GO database. The majority of ESTs (63%) belonged to this category (607 out of 971 ESTs; Figure 6). The second type was similar sequences found in the GO database with the function of those sequences annotated as "unknown." The last type of "unknown" was similar sequences found in the GO database, but only certain domains of GO annotation were complete. For example, it could only have molecular functional annotation associated with the sequence but biological process and cellular components are unknown. The last two types of sequences were combined into one "unknown category" in that particular functional domain (Figure 6). Twenty five percent of sequences was annotated in molecular function while 12% was unknown; 27% was annotated in biological process while 10% was unknown; and 27% was annotated in cellular components with 11% unknown. Among the annotated sequences, 36%, 49%, and 22% of annotated sequences were associated with unique GO terms in each of the three domains (molecular function, biological process and cellular component), respectively (Figure 6).

Both functional and sequence indexes for the three clustering methods converge around the threshold of 442 clusters. When the user-defined number of probes is fewer than this threshold value (442 clusters), the functional uniqueness of the selected probes using single linkage method is superior than that of the other two methods while average linkage is the most effective method for dimension reduction (Figure 2).

## Discussion

cDNA microarray is one of the most common microarray platforms, but it is also known to have cross-hybridization potentials. The hybridization potentials between sequences may also vary as the experimental condition changes. This changing nature and the potential of cross-hybridization could be depicted by the index developed in this study, the sequence diversity index (SDI). The magnitude of SDI decreases as the number of clusters increases; sequences are more similar within clusters as the number of clusters increases. SDI is analogous to the F-statistics. That is, SDI is the "within" variation divided by the "total" variation while the F-statistics is "within" variation divided by "between" variation. Two ancillary indexes (a functional index (UPGI) and a sequence contiguity index (CI)) were designed to evaluate the functional representativeness of the selected probes and identify the numbers of putative genes each probe potentially would cross-hybridize. These indexes aid the probe selection processes by bringing in the functional annotations of ESTs as the main goal of the microarray experiments is generally to interpret the biological significances and interactions of genes of interest. A common goal of microarray experiments is to identify co-regulated genes. This is based on the assumption that if two genes are co-expressed, they are likely to be co-regulated through the same mechanism [28]. It has been shown experimentally, at least in yeast, that combining expression data and sequence functional annotation information results in a better predictive model than using microarray expression data alone [29]. The integrated procedure in our study including both probe sequence and functional annotation allows a user-defined flexibility based on the purpose of experiments and the limitation or experimental conditions, such as different hybridization stringencies, budget limitations for numbers of probes to spot on the array, or physical size constraint of the array.

Different clustering processes mimic different scenarios of cross hybridization between sequences. Sequences from the same transcript will hybridize with each other, and this is reflected in the clusters formed using the single linkage method. In contrast, some of the sequences in the clusters formed by the complete or average linkage methods could be paralogs or alternative splicing variants of the same gene. It might be argued that if a sequence, for example Penaeidin 2, was chosen as a probe from the cluster of sequences containing different subtypes to spot on the microarray, this sequence will likely hybridize to the sequences in the same cluster, for example, Penaeidin 3a and Penaeidin 3c. The contiguity index and probe functional index developed in our study will identify the cross hybridization potential for the users. Potential cross-hybridization has become a more apparent problem for the transcriptomics community. A tool was developed to identify potential cross-hybridized probes lately [30], however, this tool is designed for species with rich genomic information. Our method provides an integrated approach for cDNA microarray design for any organisms, especially for projects with very limited genomic information.

Cross-hybridization potential between long cDNA seqeuences is harder to model than that between short (oligonucleotide) sequences. Although several studies have shown that local sequence percent identity seem to be a reasonable predictor for cross hybridization for cDNA microarray experiments [31-33], the cross-reactivity varies in a wide range (0.6 – 57% signal) even when percent identity is high and within a similar range (80–85% identity) for sequences in different gene families [33]. Currently, there is (are) no good predictor(s) to model the cross-hybridization on cDNA microarray. The similarity measurement between sequences in our study (*d*_*mi*_) could be easily replaced in the future by any good cross-hybridization predicting parameter(s) developed for long cDNA sequence hybridization. The design procedure we described here will work in the exact same fashion. In a similar manner, although the traditional hierarchical clustering algorithm with three linkage methods was used in our study, any bottom-up clustering algorithm (e.g., K-nearest means clustering) or top-down approach (e.g., principle component analysis, single value decomposition) could be easily performed, and the corresponding SDIs, UPGIs and CIs will be generated in the same way and summarized in the SDP. The performances of these different bottom-up or top-down algorithms (to group or partition the sequences) could be compared using the SDP. In brief, other distance matrix and clustering algorithms other than what we used in this study could be easily applied using our algorithm, and their performances could be evaluated quantitatively using the suite of indexes in SDP.

Annotation of functionally unknown sequences is not a trivial task itself. Gene Ontology has become a standard ontology to annotate unknown sequences. Sequence similarity search against the GO database using BLAST was used in this study. The completeness of the GO database and sensitivity/selectivity of the BLAST procedure would dictate the annotation capability. Several different approaches could potentially improve the annotation in the future. One example found in this study was Penaeidin family, a unique family of antimicrobial peptides with both proline and cysteine-rich domains that were first identified and characterized as peptides in the hemolymph of the Pacific white shrimp, *Litopenaeus vannamei *[34]. No homologous proteins are found in GO database. Future research could emphasize how to integrate other sources of knowledge (database) to enrich the functional annotation process, especially as very limited knowledge is available for marine organisms in the public domain. Different approaches to annotate the EST sequences could also be adapted. For instance, position-dependent method (such as using HMMER [35] to search against Pfam database [36,37]) could be used to search the existing database. This may increase the chance to annotate sequences with lower sequence homologies with the sequences stored in the database. The current functional representativeness of the selected probes was quantified using the unique GO terms associated with the probes among all sequences. The quantification of how representative the probes are could be modified in the future to include the hierarchical nature of the GO terms.

The integration of this cDNA probe selection procedure with the database through Marine Genomics web-based interface [27] is currently in progress, and the marine genomics community will be directly benefited, and it will be equally applicable to any organisms with limited genomic information.

## Conclusions

The sequence diversity index (SDI) was developed in this study to select probes using ESTs for designing cDNA microarrays. Two ancillary mathematic indexes (sequence contiguity index [CI] and unique probe GO index [UPGI]) were used to identify potential cross-hybridization between different transcripts (or paralogs) and to quantify biologically how representative the probes were. These three indexes were summarized in a sequence diversity plot (SDP) and were used to assist cDNA microarray probe selections for organisms without any genomic information. This method allows the user-defined number of probes to be selected for the cDNA microarray experiments. Different clustering methods balance the representativeness of the probe functional annotations and minimization of the sequence redundancies. Accordingly, different linkage methods can be used to decide between microarray designs for biomarker discovery or for functional genomics.

It is clear that sequence assembly into contigs is not necessary for microarray probe selection although it is informative to identify the relationship among sequence members within clusters based on the CI. The microarray design procedure described here could also be used for multi-species or cross-species microarray design in a scenario where the sequences with high similarity from different species cross hybridize to each other [32], but not necessary be assembled into contigs.

This method is not limited to the ESTs collected from single or multiple marine organisms. Furthermore, this method can be applied to any organisms without the complete sequenced genomes.

## Methods

### Sequence availability and pre-processing

Twenty six thousand and six hundred fifty-six (26,656) Expressed Sequence Tags (ESTs) from 14 marine species were generated and stored in a postgresSQL database through a user-friendly interface at the Marine Genomics website [27]. All the sequences are freely available to the public. One thousand and forty seven ESTs from Atlantic white shrimp (*L. setiferus*) were used in this study. Pre-processing included customized low quality filtering, poly-A tail, vector, adaptor screening, trimming, and low-complexity masking by DUST [38]. After pre-processing, 971 sequences longer than 100 bp were further analyzed (Figure 1).

### Sequence similarity comparison

All against all pair-wise BLASTN [39] was performed between these 971 ESTs. In the BLASTN result, with sufficiently large sequence lengths *q *and *n*, the statistics of HSP (high-scoring segment pairs) scores are characterized by two parameters, *K *and *lambda*. The E-value, the expected number of HSPs with score of at least *S*, given by the formula *E *= *Kqne^-λS^*, was used as the distance measurement (*d*_*mi *_in Eq (1) in results) between ESTs for cluster analysis to determine sequence redundancies. *d*_*mi *_is the distance between the i^th ^pair of sequences for a total *k*_*m *_pairs of within-cluster comparisons when *m *clusters are formed.

### Sequence redundancy reduction by cluster analyses

Hierarchical cluster analyses with three common linkage methods (single linkage, average linkage, complete linkage) were performed to reduce the redundancies among sequences.

### Two sequence indexes were used to quantify sequence diversity and contiguity within clusters

Two sequence indexes, the sequence diversity index [SDI; Eq. (1) in results] and the contiguity index [CI; Eq. (2) in results], were used throughout the sequence redundancy reduction. SDI was used to aid the number of probes to select. The within-cluster sequence contiguity (CI) is evaluated using CAP3 assembly software with default parameters [23]. Unweighted average within-cluster percent identity of the HSP segments and HSP length from BLAST results were quantified throughout the process of clusterings.

### Probe selection

To maximize the hybridization probability between the selected probe and the sequences within the cluster, the sequence has the highest similarity to all the other sequences within the cluster is selected. That is, the centroid EST, the sequence has the minimum average distance to all the other sequences within each cluster was spotted on the array.

### Sequence functional annotation using Gene Ontology (GO) terms

Functions of all 971 sequences were annotated using the functional categorizations of similar sequences stored in Gene Ontology (GO) database [40]. GO terms are commonly used for functional categorization in three domains (biological process, molecular function, and cellular component) for gene products (proteins) or nucleotide sequences. The GO terms and associated protein sequences were downloaded from the GO website [41] in the format of mySQL database [42]. The ESTs were annotated by the top BLASTX hit after blasting them against the proteins with GO terms associated in the database. The sequences with the E-value threshold set at 10^-6 ^for GO annotation are considered as similar, and they potentially share the same molecular functions, cellular components, or biological processes. The GO terms found associated with the EST sequences, if any, were recorded separately for each of the three domains. If there were multiple GO terms in any single domain (e.g., molecular function), the inverse of the number of GO terms in that domain is used for functional quantification (i.e., the traditional pie-chart summary of the functional categories of ESTs). For example, there are three molecular functional annotations (GO:0005515, GO:0004866, GO:0004867) associated with the sequence 1046, then each of them is considered 1/3 in the GO quantification for this particular sequence. Therefore, the quantification for each GO domain will sum up to the original analyzed sequence numbers at the end when we quantify the percentage of each category (n = 971).

### A functional index to quantify how representative the selected probes are

The unique probe GO index [UPGI; Eq. (3) in results] was used to quantify functionally how representative the selected probes were within EST clusters.

### Number of probes to retain using the sequence diversity plot (SDP)

Two sequence indexes (CI and SDI) and one functional index (UPGI) mentioned above were included in the sequence diversity plot (SDP) (Figure 1). Sequence similarity was measured by SDI (Eq. (1) in results), and within-cluster sequence contiguity was measured by CI (Eq. (2) in results). The unique probe GO index (Eq. (3) in results) was used to quantify functional annotation levels represented by the selected probes. This integrated information will allow user-defined flexibility of probe selection involving both sequence similarity and functional annotation.

## List of abbreviations

SDP: sequence diversity plot, SDI: sequence diversity index, CI: sequence contiguity index, and UPGI: unique probe GO index

## Figures and Tables

**Table 1:** Sequence similarities between three penaeidin sequences in a group formed by cluster analyses using the single linkage method. Percent identity (%) and sequence length (bp) of the high scoring pair from the pair wise blast results (in parentheses).

	Lset10	Lset59	Lset177
Lset10	100 (635)	88 (140)	90 (140)
Lset59	88 (140)	100 (586)	95 (141)
Lset177	90 (140)	95 (141)	100 (456)
